# Preoperative Radiotherapy with a Simultaneous Integrated Boost Compared to Chemoradiotherapy for cT3-4 Rectal Cancer: Long-Term Results of a Multicenter Randomized Study

**DOI:** 10.3390/cancers15153869

**Published:** 2023-07-29

**Authors:** Benedikt Engels, Antonino De Paoli, Elena Delmastro, Fernando Munoz, Stefano Vagge, Darius Norkus, Hendrik Everaert, Gianna Tabaro, Elisabetta Gariboldi, Umberto Ricardi, Eugenio Borsatti, Pietro Gabriele, Roberto Innocente, Elisa Palazzari, Emilie Dubaere, Marc-André Mahé, Sven Van Laere, Thierry Gevaert, Mark De Ridder

**Affiliations:** 1 Department of Radiotherapy, UZ Brussel, Vrije Universiteit Brussel, 1090 Brussels, Belgium; 2 Department of Radiation Oncology, Centro di Riferimento Oncologico (CRO)-IRCCS, 33081 Aviano, Italy; 3 Department of Radiation Oncology, IRCC Candiolo, 10060 Candiolo, Italy; 4 Department of Oncology, University of Torino, 10126 Torino, Italy; 5 Department of Radiation Oncology, IRCCS San Martino-IST Genoa, 16132 Genoa, Italy; 6 Department of Radiotherapy, National Cancer Institute, 08406 Vilnius, Lithuania; 7 Department of Nuclear Medicine, UZ Brussel, Vrije Universiteit Brussel, 1090 Brussels, Belgium; 8 Department of Radiotherapy, Institut de Cancérologie de l’Ouest, Nantes, 44800 Saint-Herblain, France

**Keywords:** rectal cancer, radiation therapy, image-guided and intensity-modulated RT (IG-IMRT), simultaneous integrated boost (SIB), chemoradiotherapy (CRT), randomized clinical trial

## Abstract

**Simple Summary:**

An international multicenter randomized trial in 174 patients with T3-4 rectal cancer was conducted by the RectumSIB consortium (located at UZ Brussel-VUB in Brussels, Belgium). In this trial, they compared standard 5FU-chemoradiotherapy (CRT) and radiotherapy with a simultaneous integrated boost (SIB) without concomitant chemotherapy in a non-inferiority design. The primary endpoint (metabolic response rate) and secondary endpoints (local control, progression-free survival, survival, pathological response and acute/late toxicity) revealed the equivalence of both treatments. Radiotherapy with an SIB is an appealing treatment strategy for patients with T3-4 rectal cancer that are too frail for chemotherapy.

**Abstract:**

Background: Preoperative chemoradiotherapy (CRT) is the standard treatment for T3-4 rectal cancer. Here, we compared image-guided and intensity-modulated RT (IG-IMRT) with a simultaneous integrated boost (SIB) (instead of concomitant chemotherapy) versus CRT in a multi-centric randomized trial. Methods: cT3-4 rectal cancer patients were randomly assigned to receive preoperative IG-IMRT 46 Gy/23 fractions plus capecitabine 825 mg/m² twice daily (CRT arm) or IG-IMRT 46 Gy/23 fractions with an SIB to the rectal tumor up to a total dose of 55.2 Gy (RTSIB arm). Results: A total of 174 patients were randomly assigned between April 2010 and May 2014. Grade 3 acute toxicities were 6% and 4% in the CRT and RTSIB arms, respectively. The mean fractional change in SUVmax at 5 weeks after completion of preoperative RT were −55.8% (±24.0%) and −52.9% (±21.6%) for patients in the CRT arm and RTSIB arm, respectively (*p* = 0.43). The pathologic complete response rate was 24% with CRT compared to 14% with RTSIB. There were no differences in 5-year overall survival (OS), progression-free survival (PFS) or local control (LC). Conclusions: The preoperative RTSIB approach was not inferior to CRT in terms of metabolic response, toxicity, OS, PFS and LC, and could be considered an available option for patients unfit for fluorouracil-based CRT.

## 1. Introduction

Preoperative 5-fluorouracil (5-FU)-based chemoradiotherapy (CRT) is the standard of care in patients with locally advanced rectal cancer. According to randomized trials, the combined treatment modality increases the pathologic complete response (pCR) rate and local control (LC) compared to RT alone, but no significant impact on overall survival (OS) or occurrence of distant metastases were observed [1,2]. Acute gastrointestinal (GI) toxicity, primarily diarrhea, is, however, common with preoperative CRT using 3D conformal RT (3D-CRT) [3]. Given the excellent LC rates achieved with preoperative CRT followed by total mesorectal excision (TME), decreasing treatment-related side effects remains an important endpoint in therapeutic decision making. 

In an attempt to lower treatment related toxicity, we introduced the concept of image-guided and intensity-modulated RT (IG-IMRT) in the preoperative treatment of rectal cancer [4,5,6]. We reported in previous studies on the synergism of improved dose distributions by IMRT and minimization of setup margins by IGRT in decreasing the irradiated volume of the small bowel, which is the major predictor of radiation enteritis [7]. As a second step to decrease toxicity, we omitted concomitant chemotherapy and delivered a simultaneous integrated boost (SIB) on the primary tumor in order to maintain oncological safety. This RT-intensified approach resulted in limited acute toxicity (1% grade ≥ 3 toxicity) and a promising 5-year LC rate of 97% in 108 cT3-4 rectal cancer patients treated in a prospective phase II trial [5,6]. 

To compare the value of this innovative strategy to the standard addition of 5-FU to preoperative RT, we conducted a multicenter randomized trial that compared preoperative intensified IG-IMRT with an SIB to preoperative standard CRT using IG-IMRT and concurrent capecitabine (NCT 01224392). The aim of this study was to demonstrate non-inferiority of a higher radiation dose compared to concomitant chemotherapy with metabolic tumor activity reduction as primary short-term endpoint. 

We report on the long-term analysis of metabolic tumor activity, disease control and patient survival of this multicenter randomized trial.

## 2. Materials and Methods

### 2.1. Inclusion Criteria

Eligibility criteria include histopathologically confirmed rectal adenocarcinoma with the inferior margin within 15 cm of the anal verge and evidence of cT3-4 disease on magnetic resonance imaging (MRI) or endoluminal ultrasound. Patients with unresectable metastatic disease at diagnosis, with an ECOG performance status > 2 and patients not deemed fit for RT, capecitabine or surgery were excluded. All patients signed their informed consent for the study protocol (see Supplementary material), which was reviewed and approved by the ethics committee of the participating centers.

### 2.2. Pre-Reatment Evaluation

Evaluation before random assignment included a complete history and physical examination, laboratory tests, digital rectal examination, colonoscopy, tumor biopsy, endoluminal ultrasound, chest–abdomen CT, MRI of the pelvis and fluorodeoxyglucose (FDG)—positron emission tomography (PET). The circumferential resection margin was anticipated on MRI as described by the MERCURY study group [8].

### 2.3. Random Assignment and Treatment

Eligible patients were randomly allocated to one of the following two treatment arms: 

IG-IMRT 46 Gy in 23 fractions of 2 Gy (5 times per week) with concurrent capecitabine 825 mg/m² twice daily, started on the first day of RT and given 5 days per week, excluding weekends (CRT arm).

IG-IMRT 46 Gy in 23 fractions of 2 Gy with an SIB of 0.4 Gy/day on the primary tumor up to a total dose of 55.2 Gy (RTSIB arm), with an equivalent dose in 2-Gy fractions (EqD2) of 57 Gy (considering α/β = 10 Gy). 

Randomization was performed centrally at the UZ Brussel, and stratification was performed by the anticipated CRM (≤1 mm vs. >1 mm) and by the required surgical procedure (abdominoperineal resection vs. low anterior resection). 

Preoperative RT was carried out with IG-IMRT using the TomoTherapy Hi-Art II System (TomoTherapy Inc., Madison, WI, USA), Elekta Infinity, Elekta Axesse or Varian Rapid Arc in 65%, 15%, 15% and 5% of the randomized patients, respectively. We included the primary tumor, mesorectum and lymph nodes along the obturator and internal iliac vessels in the clinical target volume (CTV 46 Gy). The cranial border of the CTV was set at the bifurcation of the common iliac vessels into external/internal iliac. The gross tumor volume (GTV) was defined as the macroscopic tumor delineated on the CT-scan after co-registration with PET and MRI. Based on a previous study in our department, non-uniform planning target volume (PTV) margins of 8 mm in both lateral directions, 11 mm anteriorly, 7 mm posteriorly, and 10 mm craniocaudally were applied to create the PTV46 Gy and the PTV55.2 Gy respectively [9]. The latter was only used for the RTSIB patients. The planning goals were to give at least 95% of the prescribed dose to at least 95% of the PTVs. All centers tried to minimize the volume of small bowel receiving 15 Gy or greater (V15_SB_ < 150 mL) and to keep the mean bladder dose < 21 Gy. Patients underwent daily image guidance using an integrated CT modality and were repositioned after co-registration of these images with the planning CT scan. 

### 2.4. Surgical Procedure and Pathological Evaluation

Surgery was planned within 6–8 weeks after completion of preoperative treatment. Total mesorectal excision (TME) was performed according to a standardized technique. Sphincter-sparing surgery was not an endpoint of this study, and the choice of the intended surgical procedure was made by the treating surgeon of each participating center. The resected specimen was evaluated according to the recommendations of Quirke and Nagtegaal and van Krieken [10]. Incomplete microscopical resection (R1) was defined as a CRM of ≤ 1 mm from the inked non-peritonealized surface of the specimen. The standardized five-point tumor regression grading (TRG) was used based on the work of Dworak et al. [11]. A pCR was defined as no visible microscopic disease in the primary tumor (Dworak regression grade 4).

### 2.5. Adjuvant Chemotherapy

The planned adjuvant treatment in both arms consisted of 6 cycles of capecitabine 1000 mg/m² twice daily from the evening of day 1 to the morning of day 15, every 3 weeks. Adjuvant chemotherapy was started 6–8 weeks after surgery. The conversion of the eventual temporary colostomy was recommended at the end of adjuvant chemotherapy.

### 2.6. Toxicity Monitoring and Follow-Up

Patients were seen 3 months after surgery and then every 6 months for a planned duration of 5 years. Toxicity was scored according to the NCI CTC AE version 3.0. 

### 2.7. Endpoints

The primary endpoint, metabolic tumor activity reduction, was evaluated by comparing the PET-CT at baseline with the PET-CT carried out 5 weeks after completion of preoperative treatment. All patients fasted for at least 6 h and blood glucose level was checked before image acquisition. Images were obtained 60 min after injection of 18F-FDG using a PET/CT scanner. The tumoral 18F-FDG uptake was assessed semi-quantitatively by measuring the maximal standardized uptake value (SUVmax) within a three-dimensional ellipsoidal region of interest (ROI) covering the target and carefully avoiding the urinary bladder. Special attention was made to draw the ROI in the exact same position as on the PET-CT at baseline. Uptake and reconstruction parameters were kept identical on the sequential PET-CT scans. Activity measurements were corrected for administered dose, body weight and decay. No correction for glycemia, lean body mass, or body surface was made. In order to assess tumor response to therapy, the SUVmax values in baseline (SUVpre) and reevaluation scan (SUVpost) were registered and the response index (RI), the primary endpoint, was calculated as follows:

$$RI=\frac{{SUV}_{post}-{SUV}_{pre}}{{SUV}_{pre}}\times 100$$

Secondary endpoints included acute and late toxicity, compliance to treatment arms, pCR rate, tumor downstaging, R0 resection, sphincter-sparing surgery, post-operative complications and long-term oncologic outcomes (LC, PFS and OS).

### 2.8. Quality Assurance

Participating centers were requested to participate in a dummy run procedure in order to verify the compliance with protocol contouring guidelines and treatment planning. Consistency between the different centers in the delineation of the target volumes was compared with the Dice similarity coefficient. The mean Dice similarity coefficients were 0.87 (range, 0.83–0.91) and 0.82 (range, 0.75–0.90) for delineation of the GTV and CTV, respectively. It is generally accepted that a Dice coefficient greater than 0.70 signifies good agreement [12]. In concordance, all centers had no difficulty in complying with PTV, bladder and small bowel constraints. 

### 2.9. Statistical Analysis

The primary endpoint was to demonstrate non-inferiority of RTSIB to CRT in metabolic tumor activity reduction by comparing RI values. Assuming a null hypothesis that RTSIB is inferior to CRT by at least −10% difference in RI, the so-called non-inferiority margin, a 5% type I error (one-sided), 80% power and 25% standard deviation, we calculated a sample size of 78 evaluable patients per group (SampleSize v2.04). Categorical variables were compared with Chi-square test and continuous variables were compared with the Mann–Whitney test. Actuarial LC, PFS and OS were estimated by Kaplan–Meier analysis. Forest plots were created for univariate subgroup analysis of PFS and OS respectively. Multivariable (Cox proportional hazards regression model) analyses were performed for PFS and OS, including study intervention. A value of *p* < 0.05 indicated statistical significance.

## 3. Results

### 3.1. Patient Population

**Figure 1:**
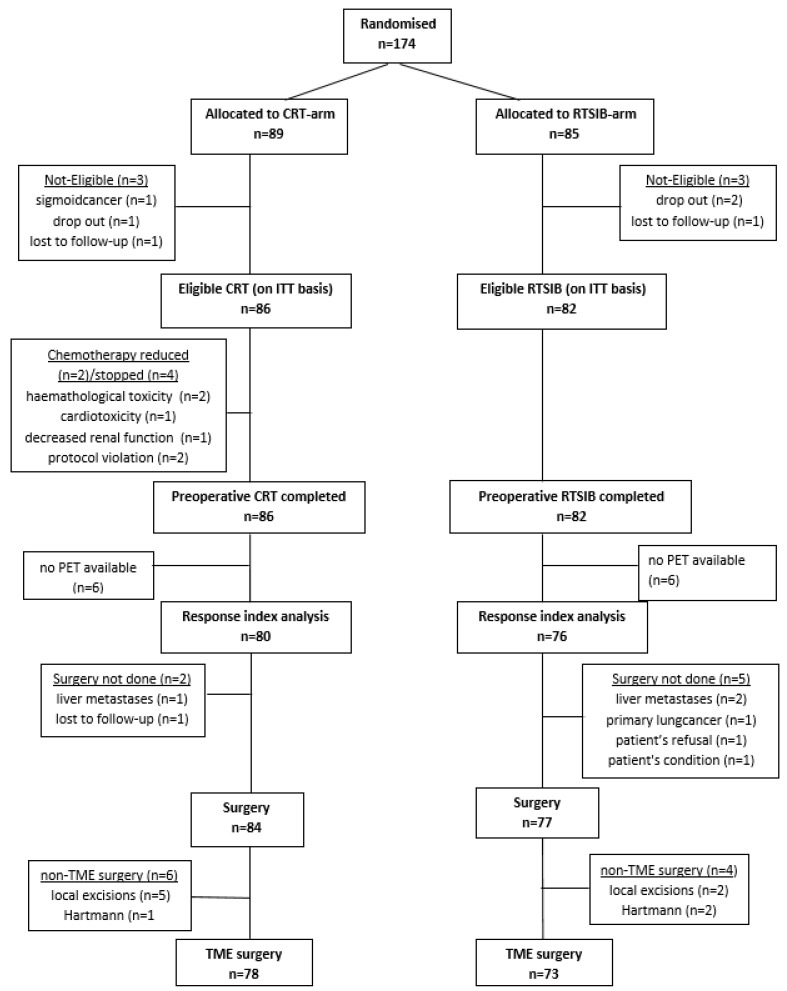
Consort diagram. Abbreviations: CRT arm, chemoradiotherapy 46 Gy in 23 fractions of 2 Gy by image-guided and intensity-modulated RT (IG-IMRT) with concurrent capecitabine 825 mg/m^2^ twice daily started on the first day of RT and given 5 days per week, excluding weekends. RTSIB arm, 46 Gy in 23 fractions of 2 Gy by IG-IMRT with a simultaneous integrated boost (SIB) of 0.4 Gy/day on the primary tumor up to a total dose of 55.2 Gy. PET, positron emission tomography. TME, total mesorectal excision.

A total of 174 patients were randomly assigned between April 2010 and May 2014 to eight institutions. Six patients were not eligible due to drop out (n = 3), loss to follow-up (n = 2) and presence of sigmoid instead of rectal cancer (n = 1). The analysis on ITT was performed on 168 eligible patients, of which 86 allocated to the CRT arm and 82 to the RTSIB arm (Figure 1). Patient characteristics were well-balanced between the 2 arms (Table 1). Thirty-six patients (42%) in the CRT arm and 37 patients (45%) in the RTSIB arm presented with an anticipated CRM of ≤ 1 mm based on MRI.

### 3.2. Treatment Compliance and Acute Toxicity

In the CRT arm, 80 patients (93%) received the full doses of capecitabine (Figure 1). RT was delivered as planned in 100% of the patients in the CRT arm and RTSIB arm. The overall rate of grade 3 acute toxicity was limited to 6% and 4% in the CRT and RTSIB arms, respectively. Table 2 lists the incidences of acute toxicities.

### 3.3. Surgery

At a median time interval of 8 weeks (range, 4–21) in both groups after completion of preoperative RT, 84 of 86 patients in the CRT arm (97.7%) and 77 of 82 in the RTSIB arm (93.9%) underwent surgical resection. Overall, seven patients were excluded from surgery due to liver metastases at restaging, patient’s refusal or poor conditions, or lost to follow-up (Figure 1). Details of surgical characteristics are listed in Table 3. The rate of sphincter-sparing surgery did not differ significantly between the two groups (75% vs. 68% respectively in CRT arm and SIB arm, *p* = 0.29). Among the 72 patients (38 CRT and 34 RTSIB) in whom an abdominoperineal resection was deemed necessary by the surgeon before randomization, sphincter preservation was achieved in 55% and 38% in the CRT and RTSIB arms, respectively (*p* = 0.15). Notably, local excision was performed in five CRT and two RTSIB patients in order to avoid the need for a permanent stoma. Anastomotic leakage of any grade occurred in 19% (n = 11) and 16% (n = 8) of the CRT and RTSIB patients respectively who underwent anterior resection, of whom six patients in the CRT arm and four in the RTSIB arm required operative reintervention. The rate of other postoperative complications was 26% in the CRT arm and 18% in the RTSIB arm (*p* = 0.22). Overall post-operative mortality within 60 days was 2% in the CRT arm and 1% in the RTSIB arm. Adherence to postoperative chemotherapy was poor, with only 35% of the patients in the CRT arm and 32% in the RTSIB arm receiving a full course of adjuvant chemotherapy. 

### 3.4. Primary Endpoint

Sequential FDG-PET images were available in 80 patients in the CRT arm and 76 in the RTSIB arm. The mean SUVmax at baseline was 15.9 (±5.8) and 15.9 (±7.9) in the CRT arm and RTSIB arm, respectively, which is significantly higher than the mean SUVmax of 6.6 (±3.9) and 6.8 (±3.6) at the re-evaluation scan (*p* < 0.01). The mean RI was −55.8% (±24.0%) in the CRT arm and −52.9% (±21.6%) in the RTSIB arm (*p* = 0.43), an RI difference of −2.9% (95%CI, −10.1% to 4.3%) (*p* = 0.43). 

### 3.5. Secondary Endpoints

The R0 resection rate was 98% in the CRT arm and 97% in the RTSIB arm. Pathologic findings are given in Table 4. An ypCR was achieved in 24% (n = 20) of the CRT group as compared to 14% (n = 11) in the RTSIB group (*p* = 0.13). Of the 31 patients with an ypCR, 26 had stage ypT0N0 and 5 ypT0Nx, the latter received local excision without lymph node dissection. Considering T-stage, tumoral downstaging from cT3-4 to ypT0-2 was observed in 51% of the patients receiving CRT and 60% of the patients who received RTSIB (*p* = 0.28). Finally, Dworak TRG 4 and 3 (no or very few tumor cells in fibrotic tissue) were reported in 49% and 45% of patients in the CRT and RT-SIB group, respectively.

### 3.6. Survival Data and Late Toxicity

**Figure 2:**
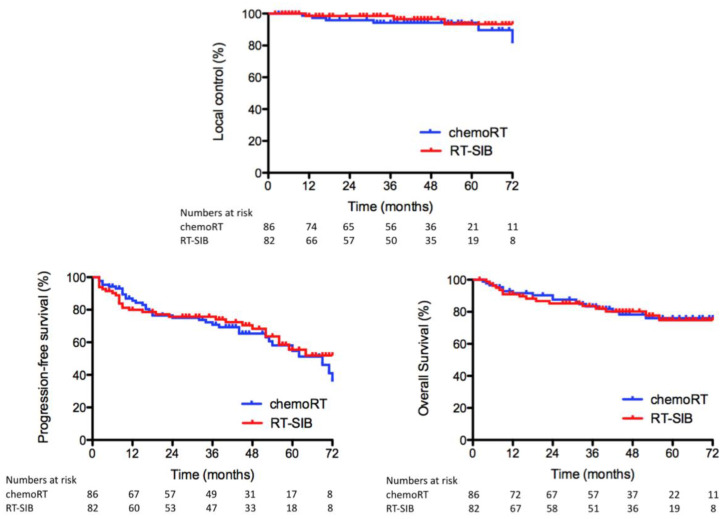
Kaplan–Meier survival curves for local control, progression-free survival and overall survival in the intent-to-treat population by study group. Abbreviations: ChemoRT, chemoradiotherapy; RT-SIB, radiotherapy with a simultaneous integrated boost.

**Figure 3:**
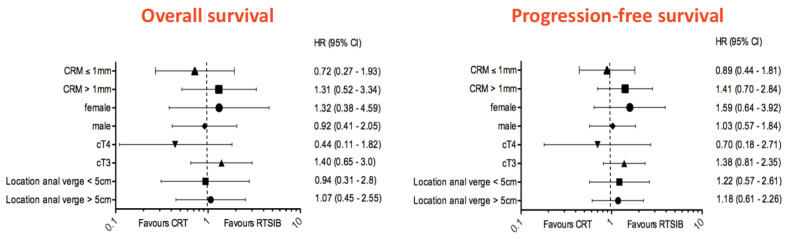
Forest plot analysis. Overall and progression-free survival of subgroups defined according to anticipated CRM, sex, T stage and tumor location. Unadjusted hazard ratios are indicated, and 95% CIs are indicated by the crossing horizontal lines. Abbreviations: CRT, chemoradiotherapy; RT-SIB, radiotherapy with a simultaneous integrated boost; CRM, circumferential resection margin.

After a median follow-up of 48 months, six patients in the CRT arm, one of which did not undergo surgery, and three patients in the RTSIB arm developed a local relapse. Of note, no local recurrences were observed in the seven patients who underwent local excision; five of these patients were complete responders, ypT0Nx, and two near-complete responders, ypT1Nx (Dworak TRG 3). We report an actuarial 5-year LC of 94.3% for CRT versus 93.4% for RTSIB (unadjusted hazard ratio (HR) for LC in the CRT arm 1.7; 95% CI, 0.46 to 6.3; *p* = 0.42). Eighteen patients (21%) died in the CRT arm and 16 patients (21%) in the RTSIB arm, with corresponding 5-year OS rates of respectively 76.1% and 74.8% (unadjusted HR 1.0; 95% CI, 0.52 to 1.2; *p* = 0.96). There were no differences between treatment arms for 5-year PFS (54.7% for CRT vs. 55.4% for RTSIB, unadjusted HR 1.2; 95% CI, 0.73 to 1.95; *p* = 0.48). Distant metastases occurred in 26% (n = 22) and 23% (n = 19) of the patients in the CRT arm and RTSIB arm, respectively. The Kaplan–Meier survival curves are given in Figure 2. Analysis of PFS and OS in subgroups defined according to tumor location, T stage, anticipated CRM and sex did not show any significant interaction with treatment group (Figure 3). Upon multivariable analysis, the presence of cT4 and ypN1-2 disease was associated with statistically significantly worse OS (*p* < 0.01), whereas cT4 (*p* = 0.01), ypN1-2 (*p* < 0.01) and Dworak grade 0–2 (*p* = 0.04) were found to be correlated with significantly impaired PFS. The absolute incidence of any grade ≥ 3 late gastrointestinal (GI) and urinary toxicity was 7% and 5% for CRT whereas 5% and 4% for RTSIB patients, respectively. Two patients died due to treatment-related toxicity; one patient in both arms died due to small bowel obstruction. Grade ≥ 3 late toxicity figures are displayed in Table 5.

## 4. Discussion

In preoperative RT of rectal cancer, either the use of effective radiation sensitizers or higher radiation dose are essentially the two approaches to enhance LC [13]. The improved dose distributions by IMRT together with a rigorous localization by IGRT enables the delivery of an SIB to the primary tumor, which produced encouraging results in terms of toxicity and LC in a phase II clinical trial [4,5,6]. This is the first phase III multicenter trial comparing preoperative intensified IG-IMRT with an SIB to standard CRT using IG-IMRT with concurrent chemotherapy followed by TME surgery in locally advanced, stage T3-4, N0-2, rectal cancer patients. After a median follow-up of 48 months, the reported 5-year LC rates of 93–94% in both arms are excellent given the number of patients with an anticipated CRM of ≤ 1 mm on preoperative MRI (42% in the CRT arm and 45% in the RTSIB arm). The 5-year PFS and OS rates in both arms are in line with the 5-year PFS and OS results of large European randomized trials, which are within the range of 56–68% and 66–76%, respectively [1,2,3]. Moreover, survival rates are also comparable to more recent published phase III trials investigating the impact of neoadjuvant CT intensification adding oxaliplatin to standard 5-FU or capecitabine-based CRT [14,15,16,17]. Interestingly, the 5-year LC (93–94%), PFS (55–55.4%) and OS (76–75%) rates are similar to those reported in the INTERACT phase III trial, comparing the addition of cisplatin to standard capecitabine-based CRT versus the CRT intensified by concomitant boost (CB) RT dose escalation to 55 Gy [18]. Even if different treatment modalities (IMRT-SIB vs. 3D-CB RT), with distinct radiation dose fractionation schedules (55.2 Gy/23 fractions vs. 55 Gy/25 fractions) and concurrent chemotherapy (IMRT-SIB alone versus 3D-CB CRT with capecitabine) were used in the present and INTERACT trial, respectively, these studies confirm the growing investigational interest in the dose escalation of preoperative RT for rectal cancer [19,20].

The major criticism of the current study may be the use of sequential FDG-PET as a surrogate endpoint for prediction of response. However, Everaert et al. (2011) [21] demonstrated sequential FDG-PET does correlate with the assessment of the response to be able to use this surrogate. The metabolic response, the primary short-term endpoint of the study, showed similar results in both arms, with a mean RI −55.8% in CRT arm and −52.9% in the RT-SIB arm (*p* = 0.43), indicating no significant difference in antitumor activity. The study was designed to have 80% power to exclude a difference in RI in favor of CRT of more than 10%. This endpoint was chosen because it has a Gaussian distribution, facilitating the non-inferiority statistics. Secondly, the choice of non-inferiority margin is critical, as it not only determines the result, but also lends scientific credibility to the study. A literature review was performed, which revealed that RI values ranged from 50% to 60% after preoperative CRT [22]. Hence, we judged that the largest difference in RI that would be clinically acceptable was 10%. As the sample size was calculated on this basis for metabolic response evaluation, the study was not sufficiently powered to reach statistical significance for the observed difference in pCR rate that was reported in 24% and 14% in CRT and RTSIB arm, respectively (*p* = 0.13). We acknowledge that pCR, and in particular SUV reduction, still remain a debated issue as reliable endpoints for phase III trials. Nevertheless, the pCR is associated with better oncological outcome, and is commonly used as early or intermediate surrogate endpoint of treatment efficacy [23]. Although apparently more favorable for the CRT arm, both pCR rates of the present study are consistent with those reported in the recent previous mentioned randomized trials, which ranged between 11.5–17.8% and 13.5–19.5% for the standard FU or capecitabine-based CRT and intensified CRT adding oxaliplatin, respectively [14,15,16,17,24]. Moreover, we observed similar tumor downstaging of 51% and 59%, and major response rates (Dworak TRG 4 and 3) of 49% and 45% for CRT and RT-SIB, respectively. These data further support the comparable activity of the two treatment modalities and appear to confirm a relationship between the intensified IMRT-SIB dose of 55.2 Gy (EqD) and tumor response [19]. Specifically, these results are well comparable to those reported from randomized trials investigating the radiation dose escalation in the neoadjuvant CRT. In the previous mentioned INTERACT trial, Valentini et al. [18] reported pCR rates of 24.4 and 23.8%, with a major response rate (Mandard TRG 1–2) of 62% and 52% (*p* = 0.003) for CB-CRT and oxaliplatin CRT intensification, respectively. Although these more promising response rates may be related to intensified treatment programs and more favorable patient selection (only stage cT3 and distal cT2 patients were included), the superior major response rate of the CB-CRT dose escalation group confirm the possible RT dose–response relationship. Similarly, in the Rectal Boost phase II randomized trial, Couwenberg et al. [25] reported a pCR or 2-year sustained clinical CR (cCR) rates of 36% and 37% for CRT with RT dose escalation to 65 Gy (15 Gy as anticipated boost) compared to standard FU-based CRT with RT dose of 50 Gy, respectively. Even if the pCR and cCR rates resulted in no difference between the two treatment arms, in this trial, too, the major response rate (Mandard TRG 1–2) was more common in the dose-escalated CRT (69%) compared to control group (45%). On the other hand, in the original work of Appelt et al. [19], too, the reported dose–response association was mainly related to TRG 1 and 2 rather than specifically to pCR. The relevance of this controversial data remains to be defined and could be a matter of further investigations. Interestingly, in the Rectal Boost trial, advanced techniques with V-MAT-IGRT were used, as reported in our study. Indeed, the incorporation of IMRT-SIB as dose escalation is currently being investigated in innovative preoperative CRT programs and more recent phase I–II trials and retrospective studies indicate its feasibility, safety, and a promising efficacy [26,27,28,29]. 

In terms of acute toxicity, no significant differences were observed between the two groups of the present trial. The 6% and 4% incidence of any grade ≥ 3 acute toxicity in the CRT and RTSIB arms, respectively is significantly lower than the 15 to 28% rate during preoperative FU-based CRT using 3D-CRT [1,2,3,14,15,24,30,31]. This remarkable difference can mainly be attributed to there being significantly less diarrhea as a result of improved small bowel sparing with IG-IMRT. Indeed, the 0–1% rate of grade ≥ 3 acute diarrhea in the current study is minimal as compared to a reported rate of 12% with 3D-CRT [3,14,15,24,31]. The 20% and 22% rate of grade ≥ 2 acute diarrhea in the CRT arm and RTSIB arm, respectively, are in line with the 23% rate reported by Samuelian et al. [32] in their retrospective experience and are consistent with our previous study that calculated a normal tissue complication probability (NTCP) for developing grade ≥ 2 acute diarrhea of 26% with IMRT [7]. Since the integration of oxaliplatin in the preoperative CRT schedule has been associated with increased rates of acute GI toxicity [14,15,24,31], one might wonder if those patients could also benefit from IMRT. RTOG 0822 was initiated as a phase II study of preoperative IMRT with capecitabine and oxaliplatin. With 18% of the patients who developed grade 3 or 4 acute diarrhea, IMRT did not seem to meet the primary endpoint of decreasing acute GI toxicity when compared to the patients treated with 3D-CRT in the RTOG 0247 trial [33,34]. In contrast to the current trial, IMRT so far does not seem to decrease the toxicity of multi-drug CRT. Nevertheless, more favorable data on toxicity were reported in phase II studies and retrospective series of IMRT-SIB with a dose range of 52.5–57.5 Gy in 22–25 fractions (2.2–2.3 Gy/fraction) and concurrent capecitabine 1650 mg/m² daily. These intensified preoperative programs using IMRT-SIB with a dose similar to the investigational arm of present trial (55.2 Gy/23 fractions) and standard dose of capecitabine demonstrated feasible and safe, with grade ≥ 3 GI toxicity in the range of 6.6–11.5%, results that are well comparable to the IMRT-SIB in this trial [26,27,28,29]. Concerning late toxicity, the 7% and 5% absolute incidence of grade 3 or greater late GI toxicity in the CRT and RTSIB arms, respectively, are comparable with the reported rate of 9% of the German CAO/ARO/AIO-94 phase III trial, where preoperative RT consisted of 50.4 Gy in 28 fractions, delivered through a three- or four-field box technique [3]. Regarding major toxicities, one patient (1%) in each arm died due to treatment-related toxicity. In comparison, Bosset and Sauer et al. reported similar rates of death from treatment complications of 1% and 2%, respectively [1,35]. IG-IMRT, with capecitabine or an SIB was associated with minimal grade 3 acute toxicity, and late toxicity appeared to be comparable to the rates after 3D conformal preoperative CRT.

We observed no difference in R0 resection between the CRT and RT-SIB group, but the high R0 rates of 98% and 97%, respectively, are remarkable considering that a large number of patients were anticipated CRM ≤1 mm (42% and 45%, respectively). Similarly, the rate of sphincter preservation did not differ significantly, but the incidence of conservative surgery for patients declared candidate to APR before randomization was substantial (55% and 38%, respectively). These data are well comparable to previous randomized trials using more conventional 3D-CRT in CRT programs [3,14,31]. Interestingly, seven complete responsive patients were selected for an organ preservation approach with local excision. A pT0Nx was confirmed in five of them, and two patients had a near-complete response (Dworak TRG 3) pT1Nx.

## 5. Conclusions

The use of preoperative IG-IMRT resulted in very low rates of grade 3 acute toxicity and similar metabolic responses in both arms. Although the use of metabolic response as surrogate endpoint has not demonstrated robust ability to predict changes in meaningful outcomes, the reported LC, PFS and OS rates in both treatment arms of the trial are intrinsically valuable to patients with rectal cancer as the current cohort harbored very locally advanced disease, with 41–46% displaying tumors < 5 cm from the anal verge, 42–45% with a threatened CRM on preoperative MRI and 82–90% with clinically node-positive disease. The question after this small randomized trial remains whether it is really relevant to omit concomitant chemotherapy, as long-course RT nowadays is not performed without chemotherapy in good-performance-status patients. Total neoadjuvant treatment has become the standard of care nowadays in high-risk patients with a good performance status. However, we do believe that by displaying similar LC, PFS and OS and toxicity rates as compared to preoperative IG-IMRT plus capecitabine, the incorporation of an SIB by IG-IMRT represents a reasonable option for frail patients who suffer cardiac comorbidities or contraindications for concurrent 5-FU or capecitabine-based chemotherapy. In these patients, the SIB-approach can still be considered a valuable, less toxic alternative to chemotherapy.

## Figures and Tables

**Table 1:** Patient characteristics at baseline.

	CRT (n = 86)		RTSIB (n = 82)		*p*-Value
Characteristics	No. of Patients	%	No. of Patients	%	
Age, years Average Range	66 48–83		65 37–87		0.67
Sex					0.63
Male	55	64	56	68	
Female	31	36	26	32	
ECOG Performance state					0.27
0	73	85	68	83	
≥1	13	15	14	17	
Distance from anal verge					0.85
<5 cm	40	46	34	41	
5–10 cm	42	49	44	54	
>10 cm	4	5	4	5	
T stage					0.35
T2	2	2	0	0	
T3—CRM > 1 mm	48	56	45	55	
T3—CRM ≤ 1 mm	31	36	28	34	
T4	5	6	9	11	
N stage					0.16
node-negative	9	10	15	18	
node-positive	77	90	67	82	
Tumor biopsy					0.93
Grade 1 adenocarcinoma	8	9	7	8	
Grade 2 adenocarcinoma	36	42	39	48	
Grade 3 adenocarcinoma	4	5	4	5	
Grade not stated	38	44	32	39	
M1	0	0	2	2	0.15

Abbreviations: CRT, chemoradiotherapy; RTSIB, radiotherapy with a simultaneous integrated boost; ECOG, Eastern Cooperative Oncology Group; CRM, circumferential resection margin.

**Table 2:** Early adverse events.

	CRT (n = 86)		RTSIB (n = 82)		*p*-Value	
Grade	2	3	2	3		
	No. of patients	No. of patients	No. of patients	No. of patients		
Gastrointestinal	36	1	35	2	0.91	(0.53)
Diarrhea	17	0	17	1	0.88	(0.30)
Enteritis (abdominal pain)	18	0	10	0	0.13	-
Proctitis	13	1	18	1	0.25	(0.97)
Urinary	7	0	11	0	0.27	-
Dysuria	4	0	10	0	0.08	-
Urinary frequency	5	0	4	0	0.79	-
Hematology	3	1	2	0	0.69	(0.33)
Anemia	0	1	1	0	0.30	(0.33)
Leucopenia	3	1	0	0	0.09	(0.33)
Thrombopenia	0	0	1	0	-	(0.30)
Other						
Hand–foot syndrome *	0	0	0	0	-	-
Radiation dermatitis	14	2	10	1	0.45	(0.59)
Vaginal mucositis	2	2	1	0	0.59	(0.16)
Overall toxicity score	43	5	46	3	0.43	(0.51)

* 5 patients in the CRT arm experienced grade 1 hand–foot syndrome; *p*-value from test grade 2 (grade 3) RTSIB vs. CRT.

**Table 3:** Surgery characteristics.

	CRT (n = 84)		RTSIB (n = 77)		*p*-Value
Characteristics	No. of Patients	%	No. of Patients	%	
Surgery					0.55
Abdominoperineal resection	20	24	23	30	
Anterior resection	58	69	50	66	
Hartmann’s resection	1	1	2	2	
Local excision	5	6	2	2	
Colostoma					0.45
Permanent	21	25	25	32	
Protective	55	65	43	56	
None	8	10	9	12	
Leakage anastomosis					0.37
Not present	74	87	69	90	
<30 days post surgery	7	12	8	10	
>30 days post surgery	4	7	0	0	
Complications	22	26	14	18	0.22
Pulmonary	3	4	2	3	
Urinary	3	4	3	4	
Fistula	4	5	1	1	
Stoma (prolapse/ necrosis)	2	2	3	4	
Abdominal wall hernia	2	2	1	1	
Perineal wound infection	2	2	1	1	
Ileus	1	1	3	4	
Peritonitis	1	1	1	1	
Heart failure	1	1	0	0	
Duration of hospital stay (in days)					
Median	11		12		
Range	4–77		4–43		
Post-operative mortality (<60 days)	2	2	1	1	0.61

**Table 4:** Surgery characteristics.

	CRT (n = 86)		RTSIB (n = 82)		*p*-Value
Characteristics	No. of Patients	%	No. of Patients	%	
Resection status					0.93
R0	82	98	75	97	
R1	2	2	2	3	
R2	0	0	0	0	
Dworak regression					0.16
Grade 0	1	1	0	0	
Grade 1	25	30	17	22	
Grade 2	17	20	25	33	
Grade 3	21	25	24	31	
Grade 4 (pCR)	20	24	11	14	
Pathological stage					0.07
ypT0	20	24	11	14	
ypTis	0	0	1	1	
ypT1	7	8	4	5	
ypT2	16	19	30	39	
ypT3	37	44	29	38	
ypT4	4	5	2	3	
Number of resected lymph nodes					
Median	12		11		
Range	2–25		1–55		
Nodal stage					0.63
ypN0	62	74	55	71	
ypN1	11	13	12	16	
ypN2	6	7	8	10	
No lymph node dissection	5	6	2	3	

**Table 5:** Late toxicity.

	CRT (n = 84)		RTSIB (n = 77)	
Grade	3	4–5	3	4–5
	No. of patients	No. of patients	No. of patients	No. of patients
Gastrointestinal	4	2	3	1
Small bowel obstruction	2	1	0	1
Stricture anastomosis	1	0	1	0
Anal incontinence	0	1	2	0
Other	1	0	1	0
Urinary	3	1	2	1
Urinary incontinence	2	0	0	0
Retention	1	0	1	0
Fistula	0	1	0	1
Other	0	0	1	0

## Data Availability

Data can be supplied upon reasonable request with the corresponding author.

## Supplementary Materials

The following supporting information can be downloaded at: https://www.mdpi.com/article/10.3390/cancers15153869/s1, Protocol S1: Study protocol of study “Simultaneous Integrated Boost Preoperative Radiotherapy for Rectum Cancer (RectumSIB)”.
