# Supplementary material for: Preoperative Radiotherapy with a Simultaneous Integrated Boost Compared to Chemoradiotherapy for cT3-4 Rectal Cancer: Long-Term Results of a Multicenter Randomized Study
Source: Cancers (Basel). 2023 Jul 29;15(15):3869. doi: 10.3390/cancers15153869 (PMC10416952; doi:10.3390/cancers15153869)
Supplement: Supplementary file 1 [file cancers-15-03869-s001.zip › cancers-2441706-supplementary.pdf]

Supplementary materials
Study protocol of study
"Simultaneous Integrated Boost Preoperative Radiotherapy for Rectum Cancer (RectumSIB)"

Primary Investigator:

Prof. Mark De Ridder, Universitair Ziekenhuis Brussel, Belgium

Collaborating centres

Universitair Ziekenhuis Brussel, Brussels, Belgium
Centro di Riferimento Oncologico (CRO)-IRCCS, Aviano, Italy
IRCCS Istituto Candiolo Centro Oncologico, Candiolo, Italy
Hospital of Aosta, Torino, Italy
IRCCS San Martino-IST Genoa, Genoa, Italy
Vilnius University, Vilnius, Lithuania
Institut de Cancérologie de l'Ouest, Nantes, France

Study Description

The investigators propose a randomized non-inferiority trial that compares preoperative Fluoro Uracil (FU)-based chemoradiotherapy to radiotherapy with a simultaneous integrated boost. In patients with T3-4 rectal cancer, the latter approach is considered preferential with regard to toxicity and cost. The metabolic response of the tumor, as assessed by 18F-2-Fluoro-2-Deoxyglucose-Positron Emission tomography (18F-FDG PET) or PET-CT, will be used as a surrogate marker of cause specific outcome

Condition or disease	Intervention/treatment	Phase
Rectal Cancer	Chemoradiotherapy<br>Radiation: Radiotherapy with boost	Phase 3

Study Design

Study Type:	Interventional
Estimated Enrollment:	156 participants
Allocation:	Randomized
Intervention Model:	Parallel Assignment
Masking:	None (Open Label)
Primary Purpose:	Treatment
Official Title:	Randomized Trial of Preoperative Radiotherapy With an Integrated Simultaneous Boost Compared to Chemoradiotherapy for T3-4 Rectal Cancer
Study Start Date:	January 2010
Actual Study Completion Date:	November 30, 2020

Arms and Interventions

Preoperative schedule

Arm	Intervention/treatment
Active Comparator: Concomitant chemoradiotherapy Radiotherapy (23 x 2 Gy) + capecitabine 825mg/m2 p.o. twice daily, excluding weekends	Chemoradiotherapy<br><br>Radiotherapy (23 x 2 Gy) + capecitabine 825mg/m2 p.o. twice daily, excluding weekends<br><br>Other Names:<br>Xeloda (capecitabine)
Experimental: Radiotherapy with boost Radiotherapy (23 x 2 Gy), with a simultaneous integrated boost up to 55.2 Gy on the primary tumor	Radiation: Radiotherapy with boost<br><br>Radiotherapy (23 x 2 Gy), with a simultaneous integrated boost up to 55.2 Gy on the primary tumor<br><br>Other Names:<br>Preoperative radiotherapy

Surgery

Surgery is performed within 5-6 weeks after completion of RT. A TME is carried out for tumors of the middle and lower third of the rectum, while a partial mesorectal excision, respecting a 5 cm distal margin is performed for tumors of the upper third of the rectum [1]. The indication of a sphincter preserving rectal resection is be made by the colorectal surgeon prior to surgery. Protective ostomies are constructed at the surgeon's discretion.

[1] MacFarlane JK, Ryall RD, Heald RJ. Mesorectal excision for rectal cancer. Lancet 1993;341(8843):457-60.

Adjuvant chemotherapy

Capecitabine 1000mg/m2 p.o. twice daily from the evening of day 1 to the morning of day 15, every 3 weeks, 6 cycles (begin 6-8 weeks after surgery).

Radiotherapeutic techniques

Preoperative schedule

FDG-PET or PET-CT is obtained prior to the start of RT (baseline) and in the fifth week after completion of RT. The PET or PET/CT acquisition protocol is performed according to the local standards. Whole body scans (base of the skull – groin) corrected for attenuation are acquired according to a similar protocol (same uptake-time, similar dose of 18F-FDG, same PET-camera). Prior to tracer injection serum glucose levels are checked.

A rectoscopy is performed to assess the tumor and measure the distance from the anal verge. During this examination, biopsies are taken, snap frozen and stored at -80°C for the translational study.

Delivery technique

A rotational IMRT technique combined by daily volumetric IGRT is proposed to reach the planning goals of the study.

GTV: delineated on fused PET-CT images, volume with a SUV > 2.5, correlated to CT or MRI imaging and endoscopy
CTV: The primary tumor, its mesentery and the lymph nodes along the internal iliac and inferior mesenteric vessels. The perineum is included if an APR is deemed necessary by the surgeon.
CTV-PTV [2]: 8 mm, except posteriorly (7mm) and anteriorly (11mm)

Planning goals

Priority	Structure	Dose specification – Constraint
1	PTV_46Gy	> 95% of volume receiving > 95% of 46Gy<br>D_{99} < 105 % of 46Gy (no boost group)
2	PTV_55.2Gy	> 95% of volume receiving > 95% of 55.2Gy<br>D_{99} < 105 % of 55.2Gy<br>conformity index < 1.5
3	small bowel	maximal dose < 50Gy<br>volume receiving more than 15Gy < 150cc
3	bladder	mean dose < 21Gy

Outcome Measures

Primary Outcome Measure

1. reduction in metabolic tumor activity [Time Frame: at baseline and at 5 to 6 weeks after neo-adjuvant therapy]

Secondary Outcome Measures

1. histological downgrading (Dworak classification) [Time Frame: after the rectum surgery]
2. number of R0, R1 and R2 resections [Time Frame: after the rectum surgery]
3. acute and late toxicity, according to the National Cancer Institute (NCI) - Common Terminology Criteria for Adverse Events v3.0 (CTCAE) [Time Frame: at baseline, every 6 months after completion of radiotherapy and then yearly until 3 years]
4. local control [Time Frame: every 6 months in the first year after completion of radiotherapy and then yearly until 3 years]
5. progression free survival [Time Frame: every 6 months in the first year after completion of radiotherapy and then yearly until 3 years]
6. overall survival [Time Frame: every 6 months in the first year after completion of radiotherapy and then yearly until 3 years]
7. quality of life [Time Frame: at baseline, every 6 months in the first year after completion of radiotherapy and then yearly until 3 years]

Statistical design

Randomized trial of non-inferiority

- Arm 1: FU-based chemoradiotherapy

- Arm 2: Radiotherapy with a simultaneous integrated boost

Primary endpoint: reduction in metabolic tumor activity.

Based on an isocontour plot of the tumor mass at 2.5 SUV the metabolic active tumor “PET volume” will be measured. Hence the metabolic volume of the tumor (PET volume times mean SUV within the 2.5 isocontour) will be calculated.

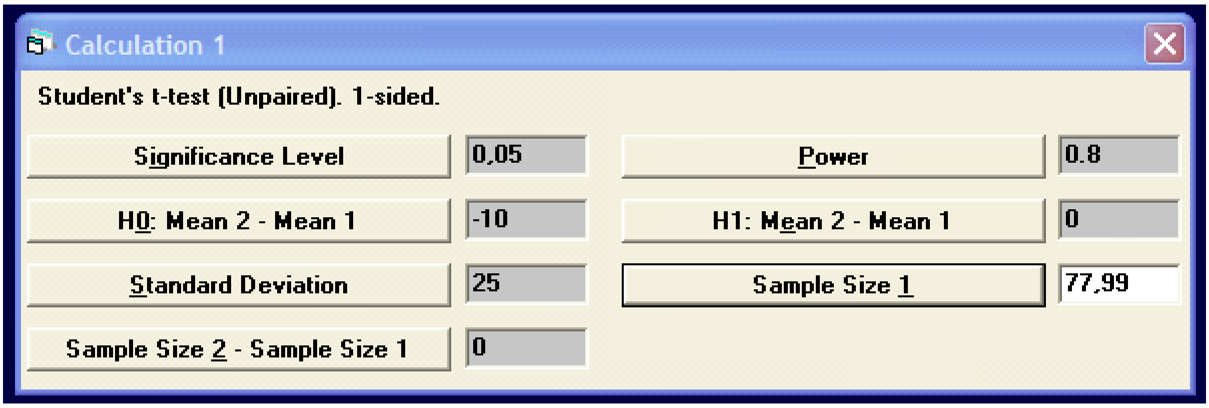

Assuming a null hypothesis that RTSIB is inferior to CRT by at least -10% difference in RI, the so called non-inferiority margin, a 5% type I error (one-sided), 80% power and 25% standard deviation, we calculated a sample size of 78 evaluable patients per group (Sam-pleSize v2.04)

Eligibility Criteria

Ages Eligible for Study: 18 Years to 95 Years
Sexes Eligible for Study: All
Accepts Healthy Volunteers: No

Criteria

Inclusion Criteria

- Histopathologically confirmed rectal adenocarcinoma with an inferior border within 15 cm of the anal verge
- The tumor has to have evidence of T3 or T4 disease on Magnetic Resonance Imaging (MRI) or endoluminal ultrasound

Exclusion Criteria

- Unresectable metastatic disease
- Eastern Cooperative Oncology Group (ECOG) performance status > 3
- Patients not deemed fit for radiotherapy, capecitabine or surgery
- Pregnant or lactating patients
- Women with child bearing potential who lack effective contraception
- Patients below 18 years old

Central Ethics Committee

Human Subjects Protection Review Board Status: Approved by CME UZ Brussel (EC-2009-253)
